# PHKA1-associated phosphorylase kinase deficiency: a monogenic disorder of exercise intolerance and myalgia

**DOI:** 10.1038/s41525-025-00527-y

**Published:** 2025-11-10

**Authors:** Rebecca L. Koch, Angie H. Fares, Benjamin T. Cocanougher, Jamie Lim, Andrea B. Haijer-Schreuder, Terry G. J. Derks, Sarah C. Grünert, Reena Sharma, Karra A. Jones, Priya S. Kishnani

**Affiliations:** 1 Division of Medical Genetics, Department of Pediatrics, Duke University Medical Center, Durham, NC USA; 2 Division of Metabolic Diseases, Beatrix Children’s Hospital, University Medical Center Groningen, Groningen, the Netherlands; 3 Department of General Pediatrics, Adolescent Medicine and Neonatology, Medical Center - University of Freiburg, Faculty of Medicine, Freiburg, Germany; 4 The Mark Holland Metabolic Unit, Adult Inherited Metabolic Diseases Department, Salford Royal NHS Foundation Trust, Salford, UK; 5 Department of Pathology, Division of Neuropathology, Duke University, Durham, NC USA

**Keywords:** Metabolic disorders, Medical genetics, Genetics research

## Abstract

Muscle phosphorylase kinase deficiency results from X-linked pathogenic variants in *PHKA1*, leading to glycogen storage disease (GSD) type IXα1 (also known as GSD IXd). As part of an international collaboration, we describe 14 previously unreported cases (12 males, 2 females; ClinicalTrials.gov NCT04454216, registered 2020-07-01). We compared our cohort to 18 cases previously reported and to an additional 16 cases identified through the National Institutes of Health All of Us Research Program. The clinical presentations highlight the predominance of myopathic symptoms on exertion and emphasize the variability in age of onset. Examination of muscle biopsies revealed glycogen accumulation and an increase in lipid droplets indicative of mitochondrial dysfunction and mitophagy. We encourage clinicians to maintain a high level of suspicion even in the setting of normal blood creatine kinase levels. Comprehensive longitudinal natural history studies remain necessary to improve disease detection, inform management guidelines, and provide a foundation for therapeutic development.

## Introduction

**Fig. 1:**
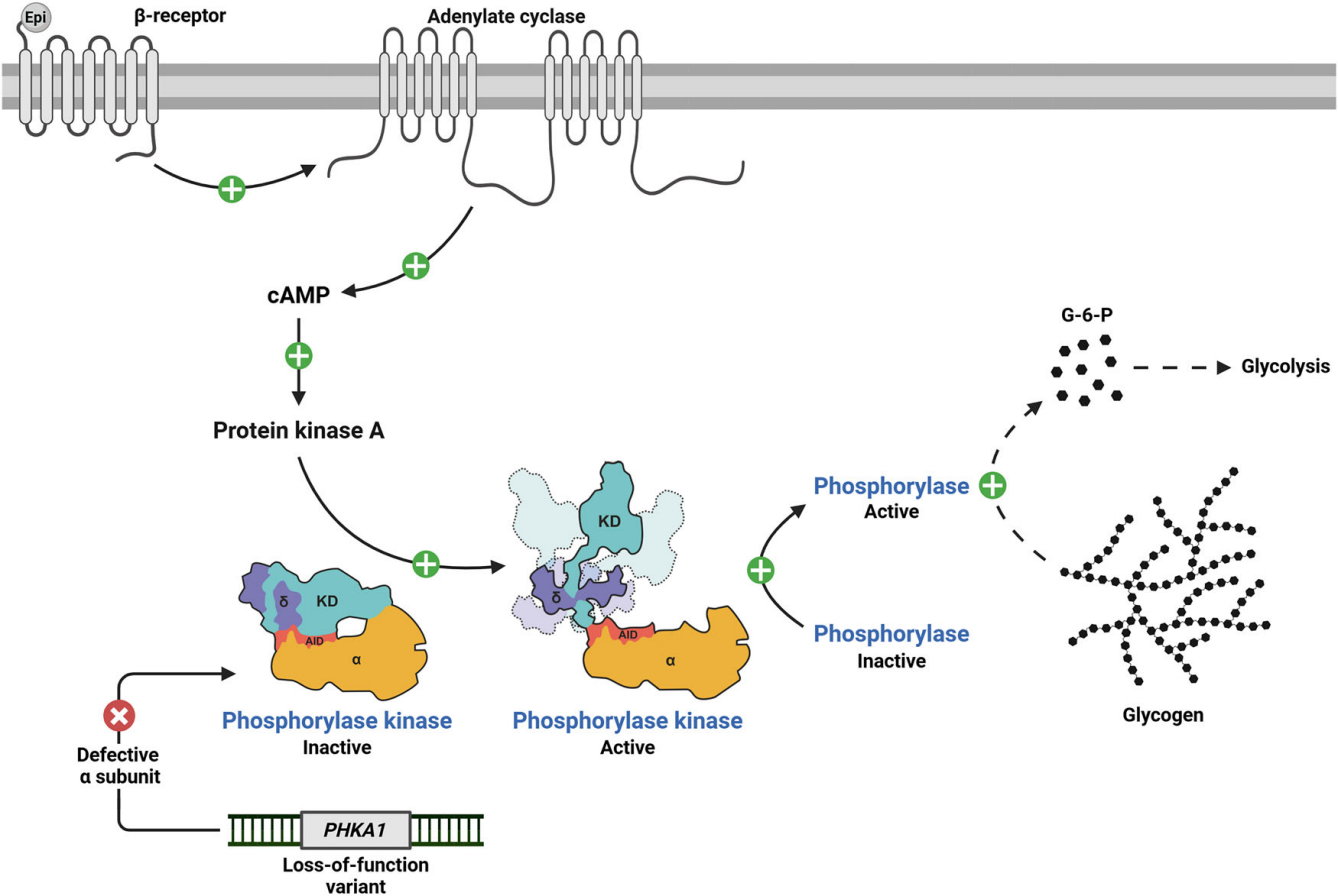
Role of phosphorylase kinase (PhK) in the muscle and the implications of pathogenic variants in *PHKA1.* A simplified schematic of glycogen synthesis in a muscle cell. When energy is needed for muscle contraction, a series of enzymatic reactions break down muscle glycogen. The regulation of this process involves stimulation of adenylate cyclase by the hormone epinephrine (Epi), which increases the cytosolic level of cAMP. The increased level of cAMP activates cAMP-dependent protein kinase which, in turn, activates PhK. PhK then activates muscle glycogen phosphorylase (myophosphorylase) which works in turn with glycogen debranching enzyme (not pictured) in a series of enzymatic actions (represented by the dashed lines) to break down glycogen into glucose-6-phosphate (G-6-P) for glycolysis. The PhK complex structure shown highlights the αγ subunit interaction, including the N-terminal catalytic kinase domain (KD) and the autoinhibitory domain (AID) of the γ subunit, and is adapted from Yang et al., *Nat Commun* (2024)^37^. In the setting of a pathogenic variant in *PHKA1* which encodes the α subunit in the muscle, the PhK complex is dysfunctional and unable to properly activate glycogen phosphorylase to initiate glycogen breakdown. Created in BioRender. Koch, R. (2025) https://BioRender.com/i13x293.

Glycogen storage disease type IXα1 (GSD IXα1, historically referred to as GSD IXd), is an ultra-rare X-linked recessive inherited disorder of carbohydrate metabolism caused by pathogenic variants in the *PHKA1* gene (OMIM: 300559, ORPHA: 715, ICD-11: 5C51.3, ICIMD: Muscle phosphorylase kinase subunit alpha 1 deficiency). GSD IXα1 is member of the GSD IX family which includes several disease subtypes affecting the liver and/or muscle based on the defective subunit (α, β, γ, or δ) of the phosphorylase kinase (PhK) heterotetramer as a result of pathogenic variant(s) in their encoding genes. The loss of a PhK subunit leads to a dysfunctional PhK complex, hindering its ability to activate glycogen phosphorylase and initiate glycogen breakdown (Fig. 1). Within the liver, the α and γ subunits are encoded by *PHKA2* and *PHKG2* and are associated with GSD IXα2 (also known as IXa) and GSD IXγ2 (also known as IXc), respectively. Alternatively, within the muscle, the α subunit is encoded by *PHKA1* and the γ subunit by *PHKG1*. The β subunit in both muscle and liver is encoded by *PHKB* and associated with GSD IXβ (also known as IXb). Neither *PHKG1* nor any of the genes encoding the calmodulin δ subunit (*CALM1*, *CALM2*, and *CALM3*) are associated with PhK deficiency in humans^1^.

As the *PHKA1* isoform is expressed primarily in skeletal muscle, GSD IXα1 is associated with dysregulated muscle glycogen breakdown and manifests with exercise intolerance, muscle cramps, myalgia, and muscle weakness^2^. There are initial reports of muscle PhK deficiency starting in the 1980s^3–13^, but to our knowledge, only 18 patients with a genetically confirmed diagnosis have been previously reported in the literature; all exhibited evidence of skeletal muscle disease, with a highly variable age of onset from infancy to late adulthood (range: 0.5–72 years)^14–29^. Given the symptoms can overlap with other muscle-related conditions, there may be a high occurrence of misdiagnosis and likelihood of underdiagnosis.

Given the limited number of cases reported, the phenotype spectrum of GSD IXα1 is incompletely described. To address this, we document the natural history and disease progression of 14 previously unreported cases that were identified clinically with deep phenotyping performed by subspecialty teams in the United States, Canada, the Netherlands, Germany, and the United Kingdom with expertise in GSD. We compare the clinical course of these cases to those previously reported in the literature, as well as analyzed the National Institutes of Health (NIH) All of Us Research Program dataset to identify additional individuals affected by the disease. Through comparison of our new cases to those previously reported in the literature and those identified through the All of Us database, we expand the number of known cases and clinical and genotypic understanding of GSD IXα1.

## Results

### Clinically identified cohort

**Fig. 2:**
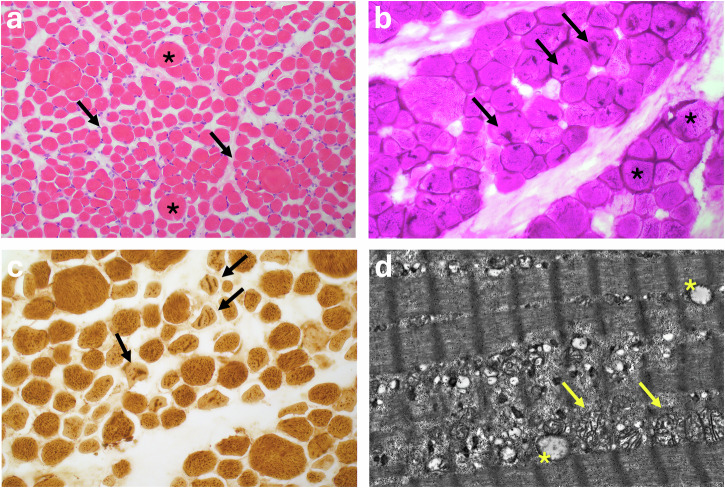
Skeletal muscle histopathologic findings from a patient with GSD IXα1. Representative sample of the left vastus lateralis from Patient 8 stained with **a** H&E (20X) showing significant fiber size variation with hypertrophic (*) and atrophic myofibers (arrows), **b** periodic acid-Schiff (40X) showing increased glycogen with an unusual sarcoplasmic linear pattern (arrows) and free subsarcolemmal glycogen accumulation (*), **c** cytochrome C oxidase (40X) showing course linear sarcoplasmic staining representing abnormalities in mitochondrial content (arrows), as well as **d** transmission electron microscopy confirming abnormal mitochondria aggregating in between myofibrils (arrows) along with large lipid droplets (*).

A total of 10 patients harbored a pathogenic or likely pathogenic variant in *PHKA1*, including 9 hemizygous males and 1 heterozygous female (Table 1). The median age at symptom onset for the pathogenic cases was 25 years (range: 0.5–49 years), and the most common chief complaints included exercise intolerance (*N* = 6), myalgia (*N* = 8), and muscle cramps (*N* = 5). Creatine kinase (CK) levels throughout follow-up were within normal limits in half of the patients (*N* = 5). Muscle biopsies were conducted in 4 participants and demonstrated variable fiber size variation with increased free and membrane-bound glycogen accumulation, rare vacuoles, and mitochondrial alterations (Supplementary Table S1; Fig. 2).

**Table 1:** Clinically identified patients with a pathogenic*/*likely pathogenic null variant in *PHKA1*

ID	Sex	Location of care	Age at onset	Age at last follow up	GSD IXα1-related chief complaints	*PHKA1* allele			Genetic testing	CK	EMG
						Variant	Location	Type			
**1**	M	Netherlands	Early childhood^a^	68	Delayed developmental milestones delay, myalgia, dysphagia	c.2806C>T (p.Arg936Ter)	Exon 25	Nonsense	WES	1.2 N	N/A
**2**	M	USA	5.5	11.3	Exercise intolerance, myalgia, fatigue	c.3334G>T (p.Glu1112Ter)^b^	Exon 31	Nonsense	WES^c^	N	N/A
**3**	M	Netherlands	9	15	Exercise intolerance, myalgia	c.2806C>T (p.Arg936Ter)	Exon 25	Nonsense	WES	N	N/A
**4**	M	USA	10	12.2	Exercise intolerance, myalgia, fatigue	c.2215C>T (p.Arg739Ter)	Exon 20	Nonsense	WES	N	N/A
**5**	M	USA	30	38	Myalgia, cramps, weakness	c.3334G>T (p.Glu1112Ter)^a^	Exon 31	Nonsense	WGS^d^	17.8 N	High frequency discharges
**6**	M	USA	31	34	Myalgia, cramps	c.892C>T (p.Arg298Ter)	Exon 9	Nonsense	Rhabdomyolysis and Metabolic Myopathy Panel^e^	4 N	N/A
**7**	M	UK	49	60	Exercise intolerance, cramps, muscle wasting	c.1152T>A (p.Tyr384Ter)	Exon 12	Nonsense	WGS	2 N	N/A
**8**	M	USA	0.5	1.2	Failure to thrive, hypotonia, dysphagia	c.1531dup (p.Tyr511LeufsTer2)	Exon 15	Insertion/ Frameshift	Rhabdomyolysis and Metabolic Myopathy Panel	N	N/A
**9**	M	USA	20	33	Exercise intolerance, myalgia, cramps, weakness	c.3244-155_3620del	Exons 30-32	Deletion	Rhabdomyolysis and Metabolic Myopathy Panel	2.7 N	N/A
**10**	F	Germany	13	35	Exercise intolerance, myalgia, cramps	c.2029del (p.Leu677Ter)	Exon 19	Deletion/ Nonsense	WGS	N	N/A

Patients are ordered by sex, age at which symptoms first presented, and type of *PHKA1* variant. Ages are provided in years. The highest creatine kinase (CK) level detected since presentation is reported and the upper limit of normal (N) was considered 310 U/L.

*N/A* not applicable/assessed, *WES* whole exome sequencing, *WGS* whole genome sequencing.

^a^An exact age of onset was not available. The patient endorsed symptoms starting in early childhood (i.e., prior to age 8 years). See the case description for more details.

^b^Variant in *PHKA1* was previously reported in the literature.

^c^A homozygous nonsense mutation in *FLG* at c.1501C>T (p.Arg501Ter), associated with ichthyosis vulgaris, was also identified and considered to be unrelated to his myopathic symptoms.

^d^A variant of uncertain significance in *RYR1* at c.1735G>A (p.Glu579Lys) was also identified and has been associated with autosomal dominant central core myopathy and congenital myopathy with fiber type disproportion.

^e^Variants of uncertain significance in *AMPD1* at c.133C>T (p.Gln45Ter) and *PYGM* at c.160T>G (p.Phe54Val) were also identified.

An additional 4 cases (3 males, 1 female) had a clinical picture consistent with muscle PhK deficiency and harbored a missense variant in *PHKA1* which was categorized as a VUS per ACMG criteria, including c.1745T>C (p.Ile582Thr), c.849C>G (p.Ile283Met), and c.1079T>C (p.Ile360Thr). The most common chief complaints included muscle weakness (*N* = 3), exercise intolerance (*N* = 2) and myalgia (*N* = 2). However, they also manifested symptoms that are less likely to be explained by the harbored VUS in *PHKA1* including bony pain (patient 13), as well as fatty liver and spleen, hypertriglyceridemia and angiokeratomas (patient 14). Further details on their clinical presentation and histology findings are provided in Supplementary Table S2.

#### Patient 1

Patient 1 is a Caucasian male who reported noticeable differences in early childhood, including not being able to turn himself over properly in the crib or keep up with his peers during physical exercise as a child. His speech always has been unclear, despite speech therapy, and this progresses during the day. He has experienced increased limitations since the age of 50 years with fatigue, diffuse myalgias, and cramping in the lower extremities and shoulders. His symptoms worsened over the course of the day and were exacerbated by physical activity; he uses a mobility scooter for assistance during walks that exceed 20 min. He reported difficulty swallowing confirmed by an otolaryngologist to be the result of reduced swallowing muscle strength. Laboratory results revealed a mild CK elevation at 365 U/L (normal limits <310 U/L). At age 66 years, WES revealed a pathogenic nonsense variant in *PHKA1* (c.2806C>T, p.Arg936Ter). No second diagnosis was identified. The introduction of MCT oil supplementation at the age of 69 years did not lead to noticeable improvement. Patient 1 receives physical therapy to address muscle strength concerns and uses analgesics as needed to manage severe myalgias.

#### Patient 2

Patient 2 is a Caucasian male who reported daily myalgia predominantly affecting his lower extremities that started at age 5 years. Whole genome sequencing (WGS) at age 6 years revealed a pathogenic nonsense variant in *PHKA1* (c.3334G>T; p.Glu1112Ter). A muscle biopsy of the left vastus lateralis displayed minimal fiber size variation, with slightly atrophic fibers exhibiting a rounded or polygonal shape, observed on hematoxylin and eosin (H&E) staining. The presence of coarse intramyofibrillar basophilia suggested abnormal mitochondria, which was further confirmed on Gomori trichrome, cytochrome C oxidase (COX), and succinate dehydrogenase (SDH) stains. Acid phosphatase staining identified rare sarcoplasmic punctate foci suggestive of vacuoles. PAS/PASD staining revealed excess glycogen in the sarcoplasm in linear aggregates and punctate forms. Oil Red O staining showed excess lipid in many myofibers. There was a normal fiber type distribution based on slow and fast myosin heavy chain immunohistochemical staining. Electron microscopy (EM) images demonstrated mostly free glycogen in between sarcomeres and occasional membrane-bound glycogen linked to autophagic vacuoles. Mitochondria were closely associated with glycogen and showed minor, nonspecific abnormalities such as enlargement, a suggestion of mitophagy, and irregular cristae (Supplementary Table S1). Myopathic symptoms have continued at age 11 years and include persistent myalgia, lower extremity cramps, exercise intolerance, and fatigue; pain was responsive to non-steroidal anti-inflammatory drugs (NSAIDs).

#### Patient 3

Patient 3 is a Caucasian male who presented at age 9 years with myalgia, fatigue, and proximal lower extremity weakness during physical activity. Whole exome sequencing (WES) revealed a pathogenic nonsense variant in *PHKA1* (c.2806C>T, p.Arg936Ter), confirmed to be maternally inherited. Physical therapy helped address the persistent myalgia and weakness. He was prescribed emulsified medium chain triglyceride (MCT) oil and controlled quantities of pre-exercise uncooked cornstarch supplementation; no symptomatic improvement was noted with these dietary interventions. Now at 15 years old, his symptoms have persisted and progressed to include exercise intolerance.

#### Patient 4

Patient 4 is a Caucasian male who was noted to have hypotonia in infancy, which resolved without intervention. Despite his early concern for hypotonia, he achieved age-appropriate developmental milestones. At age 10 years he presented to an endocrinology clinic with a 50-pound weight gain within one year in the setting of impulsive, irregular carbohydrate-rich eating behaviors. Lower extremity myalgia, exercise intolerance, and an increased bone age by 2 standard deviations compared to chronological age were noted. WES revealed a pathogenic nonsense variant in *PHKA1* (c.2215C>T, p.Arg739Ter) that was confirmed to be maternally inherited. No second diagnosis was identified. At last follow-up at the age of 12 years, he continues to experience symptoms of myalgia, fatigue, and exercise intolerance with difficulty achieving a full squat position on exam. Proximal weakness, particularly in the hip flexors and hip extensors, was also evident. He has experienced benefits from biweekly physical therapy and he takes Metformin 1500 mg per day for hyperinsulinemia.

#### Patient 5

Patient 5 is a Caucasian male who presented at age 30 years with myalgia, muscle cramps, and weakness affecting his lower extremities. An electromyography (EMG) revealed high frequency discharges and CK level was elevated (5519 U/L, normal limits <310 U/L). At age 35 years, his symptoms persisted and progressed to persistent myalgia and cramps which were more pronounced with physical activity. He also reported hand cramps and paresthesia in all four distal extremities. WGS at 36 years of age revealed a pathogenic nonsense variant in *PHKA1* (c.3334G>T, p.Glu1112Ter). No second diagnosis was identified. CK level at this time was within the normal range (97 U/L, normal <310 U/L). He was started on a high-protein diet (2–3 g/kg/day) with physical therapy, which led to clinical improvement. He was also taking Cyclobenzaprine 2.5–5 mg as needed to manage episodes of severe myalgias and reported that acupuncture and massage therapy provided significant relief.

#### Patient 6

Patient 6 is a Caucasian male that developed symptoms at 31 years of age with myalgia, muscle cramps, and fatigue on prolonged activity. Symptoms progressed to cramps in the arms, legs, and abdomen during activities of daily living. CK levels measured at age 33 years were found to be elevated (1208 U/L, normal limits <310 U/L). A rhabdomyolysis and metabolic myopathy gene panel revealed a pathogenic nonsense variant in *PHKA1* (c.892C>T, p.Arg298Ter). No second diagnosis was identified. At age 34 years, laboratory testing revealed a persistent elevation in CK levels (1236 U/L, normal <310 U/L). Patient 6 followed a vegan diet supplemented with protein powder with no reported clinical changes in muscle symptoms.

#### Patient 7

Patient 7 presented at the age of 49 years with fatigue and muscle cramping on moderate effort. He had been experiencing intermittent episodes of generalized inflammatory response following mile viral illness since the age of 30 years and had been extensively investigated by immunology and rheumatology teams with no identifiable cause. At 53 years of age, he was noted to have elevated CK levels (434 U/L) and a muscle biopsy of the right tibialis anterior was not available for review, but per report was notable for evidence of increased glycogen content (more conspicuous in subsarcolemmal vacuoles), an increase in internal nuclei, type 1 fiber predominance with no evidence of fiber type grouping, as well as fiber size variation with atrophic and hypertrophic, split, and ring fibers. There was subsarcolemmal accumulation of mitochondria including sparse ragged red fiber and several fibers with subsarcolemmal vacuoles, several of which contained debris which was further documented by modified Gomori trichrome staining. No sarcoplasmic inclusions, necrosis, basophilic fibers, or inflammation were present. Endo and perimysial connective tissue were normal. WGS detected a likely pathogenic nonsense variant in *PHKA1* (c.1152T>A, p.Tyr384Ter). No second diagnosis was identified. During his latest visit at the age of 60 years, symptoms persisted and progressed to exercise intolerance. On physical examination, muscle wasting was evident and CK levels remained elevated (635 U/L, normal <310 U/L). His neurological exam showed no weakness. An echocardiogram was performed due to mitral regurgitation murmur detected on clinical examination and it showed mild mitral and atrial regurgitation with no evidence of cardiac hypertrophy. He was advised to follow a frequent protein-rich diet with complex carbohydrates. This has greatly improved his muscle symptoms and exercise tolerance. Use of S.O.S. 25 (Vitaflo) when ill has also significantly reduced his systemic inflammatory response.

#### Patient 8

Patient 8 developed hypotonia, failure to thrive, and feeding difficulties in the first months of life. Feeding difficulties persisted despite Nissen fundoplication and the placement of a G-tube. At 7 months of age, a muscle biopsy of the right vastus lateralis (Fig. 2) was obtained followed by a rhabdomyolysis and metabolic myopathy gene panel. Biopsy showed significant fiber size variation due to rounded or polygonal atrophic and hypertrophic myofibers. Similar to patient 2, mitochondria showed abnormal aggregation or enlargement on Gomori trichrome, COX, and SDH stains. Acid phosphatase staining revealed rare sarcoplasmic punctate foci suggestive of vacuoles. PAS/PASD staining showed coarse, sometimes linear, foci of excess glycogen within myofibers. Oil Red O staining also demonstrated an increase in lipid droplet number and size. There was a normal fiber type distribution based on slow and fast myosin heavy chain immunohistochemical staining. Electron microscopy revealed increased non-membrane bound subsarcolemmal glycogen associated with mildly abnormal mitochondria showing enlargement and course or irregular cristae. On genetic testing, a likely pathogenic insertion/frameshift variant was detected in *PHKA1 (*c.1531dup, p.Tyr511LeufsTer2), leading to a termination in the reading frame. No additional diagnoses were identified. Patient 8 was 14 months old at his latest follow up visit and demonstrated persistent hypotonia and difficulty swallowing in the setting of normal CK levels (<310 U/L).

#### Patient 9

Patient 9 experienced symptomatic onset at 20 years old, initially with distal lower extremity myalgia and cramping during exertion, progressing to muscle weakness and soreness by age 25 years. His muscle pain worsened, leading to suspicion of exertional compartment syndrome at age 30 years. CK levels were elevated at 833 U/L (normal limits <310 U/L). Bilateral fasciotomies provided no relief. At age 32 years, he was assessed by a neuromuscular specialist for exacerbation of symptoms on daily walks, accompanied by persistently elevated CK levels (measuring then at 518 U/L). A rhabdomyolysis and metabolic myopathy panel revealed a deletion spanning exons 30-32 in the *PHKA1* gene (c.3244-155_3620del), classified as likely pathogenic. A muscle biopsy of the right vastus lateralis was not available for review, but the diagnosis was inconclusive per report (Supplementary Table S1). He was receiving physical therapy and was following a regular diet. At 34 years old he continues to experience persistent exercise-induced calf myalgia and cramps, with symptoms improving with rest.

#### Patient 10

Patient 10 is a female who presented at 13 years of age with significant fatigue, myalgia, and weakness following moderate to high intensity physical activity. Symptoms progressed to finger cramping and reduced finger dexterity manifesting during activities like typing, knitting, teeth brushing, and piano playing. During her twenties, she started persistent muscle cramps in her neck and back. Physical exertion, such as climbing three flights of stairs, also results in lower extremity myalgia. WES revealed a heterozygous likely pathogenic deletion creating a null variant at *PHKA1* (c.2029del, p.Leu677Ter), confirmed to be maternally inherited. X-inactivation studies were not performed as findings in blood would likely not be representative of status in muscle. Laboratory testing done at this time revealed a normal CK level (131 U/L, normal <310 U/L). She previously followed a low-carbohydrate diet for a month with no reported symptomatic improvement. Since diagnosis, she started following a protein-rich diet and receives routine physical therapy.

### All of Us cohort

As of May 2024, All of Us contained annotated genomic data on 245,388 individuals. Of these 245,388 individuals, we identified 76 individuals with rare frameshift, in-frame deletions, start-loss, or stop-gain variants in *PHKA1*. These variants meet criteria to be classified as pathogenic or likely pathogenic per American College of Medical Genetics criteria and are consistent with a diagnosis (hemizygous males; 16/76) or carrier status (females; 60/76) for GSD IXα1 (Supplementary Fig. S1-S3). No individuals carried a formal diagnosis of GSD IXα1 (or related terminology) in available medical records from the All of Us dataset.

Next, we investigated if there was evidence of the GSD IXα1 phenotype present in the All of Us cohort data. To do so, we created a concept set in the All of Us workbench that included previously reported phenotypes of GSD IXα1, including exercise intolerance, muscle pain, muscle atrophy, fatigue, and muscle cramps (Supplementary Fig. S4). This GSD IX concept set identified 1697 occurrences of potentially relevant GSD IXα1-related phenotypes among the 76 individuals. The conditions were reviewed and non-relevant diagnoses, such as “abdominal pain”, were removed. This left 1245 relevant electronic health record (EHR) entries identified in 46% of the cohort (35/76).

Of the16 affected males in the cohort, 5 had diagnoses documented that were relevant to GSD IXα1. These diagnoses included multiple joint pains (4/5) and chronic pain (2/5). No diagnoses related to muscle weakness or exercise intolerance were reported. For carrier individuals, 30 of the 60 individuals had GSD IXα1-related phenotypes. This included pain (28/30), chronic pain (18/30), and muscle weakness (6/30). The rate of chronic pain in GSD IXα1 (60%) is higher than the 3% prevalence observed across the All of Cohort with genome data available (8157/245,388).

### Cases of GSD IXα1 reported in the literature

In total, 18 male patients with GSD IXα1 were identified through our literature search (Table 2). The median age at onset was 31 years (range: 0.5–72 years) and the most common chief complaints included myalgia (*N* = 6/18), muscle weakness (*N* = 5/18), exercise intolerance (*N* = 4/18), and elevated CK levels (*N* = 2/18) (Table 2). One patient (L14) harbored the same c.3334G>T (p.Glu1112Ter) variant in *PHKA1* detected in patients 2 and 5 in our cohort. Muscle pathology findings were reported for 16 of the 18 cases (Supplementary Table S1); the biopsies demonstrated increased glycogen accumulation on PAS stain (*N* = 15/16) and variation in muscle fiber size on hematoxylin and eosin (H&E) stain (*N* = 5/16).

**Table 2:** Previously reported cases of GSD IXα1

ID^a^	Sex	Age at onset	Age at last follow up	GSD IXα1-related chief complaints	*PHKA1* variant			CK	EMG	Reference
					Variant	Location	Type			
L1	M	0.5	56	Myalgia	c.695del (p.Ala232ValfsTer11)	Exon 7	Deletion/ Frameshift	10 N	Normal	Wuyts et al. ^28^
L2	M	6	18	Exercise intolerance	c.896A>T (p.Asp299Val)	Exon 9	Missense	6 N	Myopathic changes	Clemens et al. ^29^ and Burwinkel et al. ^17^
L3^b^	M	“Childhood”	13	Muscle weakness	c.3670_3924del255^e^	Exons 29-30	Deletion	2 N	NA	Huang et al. ^19^
L4	M	“Childhood”	50	Exercise intolerance	c.667G>A (p.Gly223Arg)	Exon 7	Missense	2 N	NA	Ørngreen et al. ^23^
L5	M	15	16	Myalgia	c.3579_3580insT (p.Ser1194Ter)	Exon 32	Insertion/ Nonsense	“Elevated”	NA	Munekane et al. ^22^
L6^c^	M	15	25	Muscle weakness	c.3246T>A (p.Cys1082Ter)	Exon 30	Nonsense	“Elevated”	Normal	Li et al. ^20^
L7	M	15	28	Exercise intolerance	c.3498+1G>C (p.?)	Intron 31	Splice site	3 N	Mild myopathic changes	Bruno et al. ^16^
L8^d^	M	16	53	Myalgia	c.1360A>G (p.Ile454Val)	Exon 14	Missense	3.6 N	Compatible with moderate muscular damage in lower limbs	Picillo et al. ^24^
L9	M	17	17	HyperCKemia	c.1394del (p.Thr464fsTer486)^f^	Exon 14	Deletion/ Frameshift	5 N	Mild myopathic changes	Echaniz-Laguna et al. ^18^
L10	M	31	31	Muscle weakness	c.3297+5G>A (p.?)	Intron 30	Splice site	30 N	Myopathic changes	Huang et al. ^19^
L11	M	32	39	Myalgia	c.1293del (p.Thr432LeufsTer14)	Exon 13	Deletion/ Frameshift	1.7 N	Myopathic changes	Preisler et al. ^25^
L12	M	40	41	Chest distress	c.1533T>A (p.Tyr511Ter)	Exon 15	Nonsense	55 N	Myopathic changes	Huang et al. ^19^
L13	M	40	55	Exercise intolerance, myalgia	c.2594del (p.Lys865ArgfsTer15)	Exon 23	Deletion/ Frameshift	20 N	Normal	Bisciglia et al. ^15^
L14	M	46	64	Gait disturbance	c.3334G>T (p.Glu1112Ter)	Exon 31	Nonsense	2 N	Myopathic changes	Wehner et al. ^27^
L15	M	64	66	Progressive muscle weakness, myopathic face, dysarthria, chewing and swallowing difficulties	c.1989_1990delinsAAGTTGCTCGTGATCTAAA (p.Tyr663Ter)	Exon 19	Nonsense	N	Typical myotonic discharges	Wang et al. ^26^
L16	M	64	69	HyperCKemia	c.695del (p.Ala232ValfsTer11)	Exon 7	Deletion/ Frameshift	5 N	Normal	Preisler et al. ^25^
L17	M	71	78	Difficulty with climbing stairs	c.915A>T (p.Lys305Asn)	Exon 9	Missense	1.05 N	Rapid recruitment with abundant polyphasic motor unit potential, fibrillation, and positive sharp waves	Mori-Yoshimura et al. ^21^
L18	M	N/A	33	Myalgia, fatigue	c.586G>A (p.Glu196Lys)	Exon 6	Missense	“Elevated”	NA	Andersen et al. ^14^

We conducted a literature search in MEDLINE and PubMed to identify articles with patients with GSD IXα1 published through May 2024. Search terms included “glycogen storage disease type IX,” “glycogen storage disease type IXα1”, “glycogen storage disease type IXd”, “phosphorylase kinase α1”, “*PHKA1* gene”, “muscle PhK deficiency”, “GSDIXd”, and “GSDIXα1” which were used in various combinations and permutations across the databases. Each individual published case was assigned an ID with a preceding “L” for “literature”. Patients are ordered by age at which symptoms first presented and age at last follow up. Ages are provided in years. Creatine kinase (CK) upper limit of normal (N) was considered 310 U/L.

*EMG* electromyography, *N/A* not applicable.

^a^An additional two individuals (*N* = 1 sister of male proband, *N* = 1 sex unknown) with a *PHKA1* variant have been reported but did not meet our inclusion criteria due to limited patient details being available^15,52^.

^b^L3 also harbors a pathogenic mitochondrial variant in *MT-TL1* at m.3243A>G with a heteroplasmy of 87.1%.

^c^L6 also harbors a concomitant pathogenic heterozygous missense variant in *KCNJ2* at c.899G>C, consistent with Andersen-Tawil syndrome.

^d^L8 harbors compound heterozygous missense variants in *GAA*, c.784G>A (pathogenic) and c.956-6T>C (VUS), associated with Pompe disease.

^e^The c.3670_3924del255 variant is reported to skip exons 29 and 30 of *PHKA1*. The nucleotide numbering could not be confirmed and is written here as it was originally reported^19^.

^f^The c.1394del variant is reported to introduce a frameshift and cause a premature stop codon in exon 14 of *PHKA1*. The nucleotide numbering could not be confirmed and is written here as it was originally reported^18^.

## Discussion

GSD IXα1, also known as GSD IXd, is an ultra-rare muscle GSD caused by hemizygous, or rarely heterozygous, loss-of-function variants in *PHKA1*. In this multicenter international study representing patients from the United States, the Netherlands, Germany, the United Kingdom, and Canada, we present 10 new cases with a pathogenic or likely pathogenic variant in *PHKA1*, as well as detail the clinical courses of 4 additional cases with a VUS in *PHKA1*. Using the NIH All of Us resource, we identified an additional 16 individuals with pathogenic/likely pathogenic hemizygous *PHKA1* variants. By comparing our clinically identified cases with those from NIH All of Us and prior cases reported in the literature, we were able to distill the key clinical features in GSD IXα1: persistent exercise intolerance with muscle pain and variable muscle weakness.

To date, 18 cases of GSD IXα1 have been reported. Our additional 10 cases with pathogenic/likely pathogenic variants in *PHKA1* and 4 cases with a VUS emphasize the high prevalence of muscle pain and cramping, especially in a limb-girdle distribution, in the setting of moderate intensity exercise. The muscle-specific form of PhK is crucial for breaking down muscle glycogen to provide energy for muscle contractions and thus deficiency of functional muscle PhK can lead to exercise intolerance, muscle cramps, myalgia, and weakness, explaining the clinical presentation of our cohort. Proximal muscles of the lower extremities were unanimously affected in patients of our cohort, with all patients reporting pain in these muscle groups (except for patient 8, who is currently 1.2 years old). Interestingly, when compared to the lower extremities, the upper extremities and other muscle groups (arms, abdominal muscles, shoulders) were less commonly involved and the number of muscles affected did not necessarily correlate with the overall severity or disease morbidity. To improve clinical recognition and comparability across future studies, it is important to develop standardized tools for assessing exercise intolerance in GSD IXα1. Quantitative measures such as the 6-minute walk test (6MWT), forearm exercise test, and cycle ergometry have been effectively used in disorders like McArdle disease (GSD V) and mitochondrial myopathies to assess functional capacity and glycolytic integrity^30–32^. For instance, Vissing & Haller demonstrated a marked improvement in exercise tolerance via cycle ergometry following oral sucrose ingestion in McArdle disease^33^. Similar to what is recommended for other muscle GSDs, validated patient-reported outcome measures (PROMs) and wearable activity tracking could further capture exertional symptoms and fatigue in a standardized way^34–36^. Due to similarities in disease presentations, these approaches would potentially enable more accurate phenotyping, longitudinal monitoring of disease burden, and facilitate therapeutic trials by providing reliable outcome measures.

**Fig. 3:**
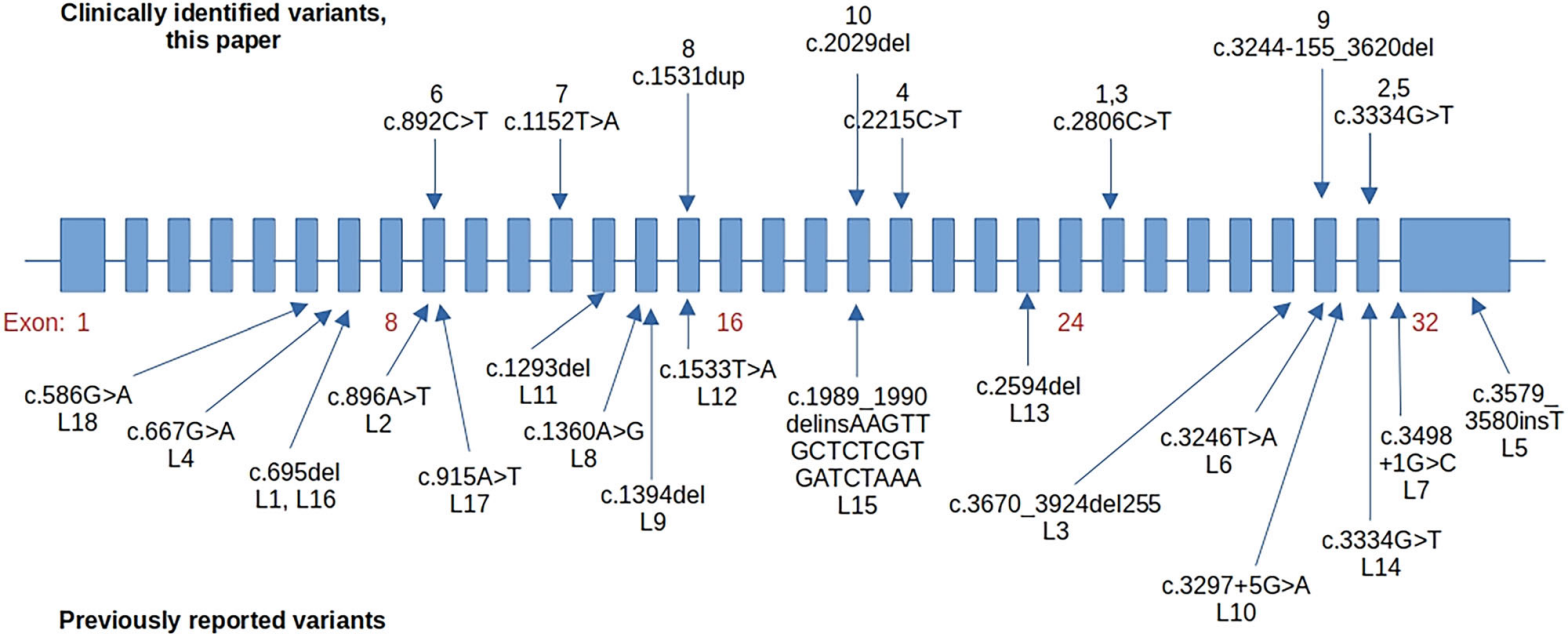
Variants in *PHKA1* along the gene from the clinically identified cohort (this paper) compared to variants previously reported in the literature. Variants in *PHKA1* correspond to transcript NM_002637.4.

Additional research is needed to better define genotype-phenotype relationships in this disease. In our clinically identified cohort, the majority of patients with a pathogenic/likely pathogenic variant in *PHKA1* harbored a nonsense null variant, while the remaining patients had a deletion or insertion variant creating a frameshift in the genetic code and a termination in transcription. The variants fell along the length of the gene (Fig. 3). Patients 1 and 3 shared the same pathogenic variant *PHKA1* (c.2806C>T, p.Arg936Ter) in exon 25; patient 3 suffered from exercise intolerance and myalgia in childhood in the setting of a normal CK level while patient 1 presented with delayed developmental milestones, myalgia, and dysphagia in childhood with CK levels 1.2× the upper limit of normal. Additionally, patient 2, patient 5, and L14 shared the same pathogenic variant (c.3334G>T, p.Glu1112Ter); patient 2 presented in early childhood with exercise intolerance, myalgia, and fatigue, whereas patient 5 presented with myalgia, cramps, and weakness in early adulthood with CK levels 17.8× the upper limit of normal, and L14 exhibited gait disturbance in middle adulthood with CK levels 2× the upper limit of normal. These findings underscore the need for more comprehensive investigations and genetic testing for individuals exhibiting exercise intolerance and myalgia that cannot be explained by other diagnoses and where a clinician has a high index of suspicion for a diagnosis of GSD IXα1.

*PHKA1* is located on the long arm of chromosome X (Xq13.1) and extends over ∼135 kilobases (GRCh38.p14). *PHKA1* encodes the alpha subunit of the large, tetrameric complex PhK, composed of four alpha (α) subunits, four beta (β) subunits, four gamma (γ) subunits, and four delta (δ) calmodulin subunits. The structure of the full complex was recently solved by cryo-electron microscopy^37,38^, which revealed the location of the α subunit as a bridge between the β subunit and the γδ subunits and suggested a clamping activation mechanism. The authors emphasized calmodulin-responsive domains in the α subunit which will be important sites for future functional testing. Nevertheless, loss-of-function variants in *PHKA1* render the α1 subunit – and thus the larger PhK complex – dysfunctional. Given that PhK functions as a protein complex, testing the activity of an individual subunit is not feasible. However, enzyme testing remains an important tool to provide supportive evidence of PhK complex dysfunction, particularly in cases where the variant identified in *PHKA1* is a VUS. All of the missense variants in our cohort were classified as VUSs per ACMG criteria, as no qualifying functional data from our patients existed to establish a loss-of-function phenotype. Performing enzyme testing is experimentally feasible and is important for reclassification of VUSs – including missense variants – observed in patients.

Our cases spanned a diverse age spectrum, supporting the emergence of a group with early-onset disease manifestation. Patient 1 and 2 were the youngest in our cohort to report myopathic complaints with onsets in early childhood (i.e., before age 8 years). Moreover, physical activity intolerance was detected at 2.4 years in patient 11. Even in patients with the same variants, such as patients 2 and 5 in our cohort and previously reported case L14, there was a wide variation in disease onset (5.5, 30, and 46 years, respectively). There was additional variability observed in the functional and blood biomarker findings. EMG results – both from our clinically identified cohort and those reported in the literature – were inconsistent, with some having normal results and others exhibiting myopathic changes. Additionally, CK levels were commonly used in clinical investigations for monitoring myopathic disease; CK elevation was only observed in 5/10 patients in our cohort, compared to 17/18 previously published cases. We encourage clinicians to maintain a high level of disease suspicion even in the setting of normal blood CK levels and EMG findings.

The lack of CK elevation may be related to the lack of myofiber degeneration/necrosis seen in the muscle biopsies. In a normal state, PhK is a key regulator of glycogen metabolism, which is crucial in skeletal muscle for sustained performance during prolonged activities. Under the effect of epinephrine, cytosolic cAMP levels rise, activating cAMP-dependent protein kinase, and then PhK phosphorylates (activates) myophosphorylase^39^. This process catalyzes the sequential cleavage of glycogen chains, releasing glucose-1-phosphate, which is subsequently converted to glucose-6-phosphate to meet muscle energy demands (Fig. 1A)^32^. During moderate to high-intensity activities, the amplitude of the mismatch in the supply–demand of ATP becomes apparent in case of PhK deficiency, leading to symptoms of exercise tolerance, fatigue, and myalgia. We postulate that the muscle fibers in individuals with GSD IXα1 may be damaged and not working efficiently, causing symptoms such as declining exercise intolerance, weakness, myalgia, or cramping, but not full degeneration/necrosis resulting in release of high levels of CK into the bloodstream. This can be compared to another rare muscular inherited disorder of carbohydrate metabolism with close enzymatic proximity: McArdle disease, associated with myophosphorylase deficiency. In McArdle disease, patients have a similar disease presentation with muscle pain in the setting of moderate to high intensity activity^34^, but there is muscle fiber damage which leads to blood CK elevation. Interestingly, the severity of myopathy observed in our cohort is less pronounced when compared to McArdle disease, including the incidence of rhabdomyolysis which is a key feature of McArdle disease but has not been observed in GSD IXα1. Given that PhK is responsible for activating myophosphorylase, these clinical differences suggest that there are alternative mechanisms of activating myophosphorylase in the setting of deficient muscle PhK. Alternatively, there may be a threshold at which a low residual level of functional PhK can maintain enough glycogen breakdown to avoid myofiber degeneration. Nonetheless, applying the management principles published for McArdle disease^28^ will likely be valuable in managing patients with GSD IXα1.

It is important to differentiate GSD IXα1 from other muscle-energy GSDs which have a block in glycolysis and are characterized by muscle pain, exercise intolerance, and susceptibility to fatigue. Muscle-energy GSDs include McArdle disease (GSD V, deficiency of myophosphorylase encoded by *PYGM*), GSD VII (Tarui disease, deficiency of phosphofructokinase-M encoded by *PFKM*), IXβ (deficiency of PhK due to β subunit defect encoded by *PHKB*), X (deficiency of muscle phosphoglycerate mutase encoded by *PGAM2*), XI (deficiency of lactate dehydrogenase A encoded by *LDHA*), XII (deficiency of fructose-1,6-biphosphate aldolase A encoded by *ALDOA*), XIII (deficiency of β-enolase encoded by *ENO3*), and phosphoglycerate kinase deficiency (encoded by *PGK1*). Though there are overlapping clinical features, the age of onset, severity of symptoms, and involvement of other organs varies between the different types of muscle-energy GSDs^40^. In the absence of reliable blood biomarkers for GSD IXα1, it may be worth exploring the utility of urine glucose tetrasaccharide (Glc4) which is a biomarker of other muscle GSDs including GSD II (Pompe disease). Nonetheless, the absence of a specific biomarker further highlights the importance of maintaining a high index of clinical suspicion and utilizing genetic testing technologies to obtain a diagnosis.

Muscle biopsy can be used for direct assessment of muscle architecture, glycogen content, and muscle PhK enzymatic activity^2^. It is critical that any muscle biopsy is expert-guided and taken from an affected muscle. Histological findings of patients in our cohort who received a muscle biopsy were similar to previously reported changes in patients with GSD IXα1, including increased fiber size variation and excess glycogen deposition. However, coarse glycogen accumulation in an unusual linear and punctate pattern detected in patients 2 and 8 with mitochondrial alterations and increased lipid have not been previously discussed in GSD IXα1. The linear pattern of glycogen accumulation was noted on both PAS and electron microscopy where it was found in between sarcomeres, sometimes membrane-bound. An accumulation of subsarcolemmal free glycogen was also seen. While identified mitochondrial abnormalities were mild and nonspecific (enlargement, aggregation, course cristae formation, and possible mitophagy), these changes were found alongside abnormal glycogen in a pattern similar to what has been described in Pompe disease muscle biopsies^41,42^. Also seen in associated with excess glycogen was definitive autophagic pathology in patient 2. Similar findings indicative of autophagic vacuolar pathology were seen in patient 11 (Supplementary material) who harbored a VUS in the *PHKA1* gene. Type I fiber predominance observed in L8 and L11 was only seen in a few of our cases (patients 7 and 9).

The *PHKA1*-deficient I-strain mouse model, further developed into a PHKA1-deficient I/LnJ model, offers potential insights into the pathophysiology of GSD IXα1^43,44^. When quantified over training periods of either 1, 2, or 5 weeks, I/LnJ mice ran significantly less time/day and distance/day than age matched C57/Bl6 wild-type mice. After 5 weeks, C57/Bl6 mice demonstrated an increase in endurance resulting from aerobic training, whereas this physiological adaptation was not present in I/LnJ mice. However, the transcriptional expression of key enzymes related to glucose transport and glycolytic flux showed no significant differences compared to wild-type mice, implying the involvement of non-glycolytic mechanisms in muscle function^44^.

Given the limited number of reported patients, we turned to the NIH All of Us cohort which is a diverse precision medicine dataset and could provide a glimpse into disease manifestations for patients without a formal diagnosis. The All of Us cohort underscores a higher caseload than documented in the literature, which highlights the challenges inherent in diagnosing ultra-rare disorders, particularly when the symptoms can be mistaken for more common disorders, such as myalgia and fatigue in the setting of a normal CK. Assuming that the All of Us cohort is broadly representative of the 333.3 million Americans (population data from 2022 United States Census Bureau report), these numbers can be used to estimate the number of affected and carrier individuals in the United States. We identified 16 affected individuals out of 94,756 individuals assigned male at birth with available WGS short read data (Supplementary Fig. S2) which gives a disease frequency of 0.00016885474. Multiplied by the population of males in the United States (165.28 million) gives an estimate of 27,908 affected males in the United States. Using the same logic, the frequency of carriers is 0.00041219265 (60/145,563) (Supplementary Fig. S3); in the United States, we estimate the number of carriers to be 69,248. Notably, in both the affected and carrier subset, direct or indirect evidence of chronic pain was present. Given the multifactorial nature of pain, it is unlikely that any of these patients would be referred for genetic evaluation. There are likely many genetic conditions, such as GSD IXα1, that remain overlooked and may contribute to the development and severity of pain. Further investigations to quantify and characterize the nature of the chronic pain within the context of related muscle symptoms are warranted. Additionally, the quality and responsiveness to therapy is an area that needs further exploration in GSD IXα1. Interestingly, no diagnoses related to muscle weakness or exercise intolerance were reported in our All of Us cohort. This could potentially be due to lack of standardized vocabulary used in healthcare and this health database; utilization of Human Phenotype Ontology (HPO) or a similar system should be encouraged to ensure a standardized vocabulary of clinical features is used.

There is conflicting evidence on the role of diet and carbohydrate supplementation prior to physical activity in GSD IXα1^14,23,25^. Based on evidence from McArdle disease, the absence of myophosphorylase necessitates heavy reliance on bloodstream circulating glucose during the first minutes of physical activity^32^. Then, a “second wind” phenomenon in McArdle disease is characterized by the ability to increase work output after ~10 min of activity due to increased availability of free fatty acids and circulating blood glucose^45^. Therefore, consuming controlled quantities of carbohydrates (i.e., sucrose) prior to physical activity may enhance exercise tolerance prior to the onset of the “second wind” phenomenon in McArdle disease^33^. Further studies are required to evaluate the occurrence, or lack thereof, of a second wind phenomenon in GSD IXα1, and if supplemental carbohydrate prior to physical activity results in any benefits. In our cohort, patient 3 was reported to have consumed controlled quantities of pre-exercise uncooked cornstarch supplementation, and no symptomatic improvement was noted. A modified ketogenic diet has also been proposed as a potential therapeutic option in McArdle disease and patients reported some symptom improvements after following the diet for 3 weeks^46^. In our cohort, patient 10 followed low-carbohydrate diet for a month with no reported symptomatic improvement; however, the macronutrient distribution of this patient’s diet was not available and it’s unclear if this diet induced ketosis. Lastly, compared to a carbohydrate-rich diet, a protein-rich diet has been shown to be suboptimal in McArdle disease^47^. In our cohort, 3 patients (patient 5, 7, and 10) followed a high-protein diet, and 2 of them reported symptomatic improvements. Yet, controlled, randomized crossover studies are needed to determine the benefits, if any, of a high-protein diet in GSD IXα1. Despite the observations and learnings from McArdle disease, it remains unclear how these findings translate to GSD IXα1, warranting further studies to elucidate the potential effects of diet and alternative energy sources on this condition. Implementing a multidisciplinary, patient- and family-centered approach upon diagnosis is crucial, with a focus on preserving and enhancing muscle strength through tailored physical therapy and dietary interventions.

While deeper genotype-phenotype correlations remain necessary for better disease characterization, the findings of this study expand our understanding of GSD IXα1. By more than doubling the cases previously reported, we show that GSD IXα1 is a monogenic disorder characterized by persistent exercise intolerance, fatigue, and chronic myalgia, with variability in age at presentation. Yet, it is important to acknowledge certain limitations. First, due to its retrospective design, the quality and completeness of the data are contingent upon the accuracy of existing records. Additionally, the international collaborative nature of the study introduces variability in data collection and management practices across different service providers, particularly because there are no published clinical practice guidelines for GSD IXα1. Prospective longitudinal studies would offer a more robust approach to elucidating the natural history of GSD IXα1, allowing for the aggregation of clinical presentations across various age ranges and expanding the scope of analysis. Additionally, as GSD IXα1 is an X-linked recessive disorder predominantly affecting males, the presence of female cases in our study raises questions about the underlying mechanisms leading to disease expression. In our reported female cases, the exact mechanism leading to GSD IXα1 expression was not specified, and X-inactivation studies were not conducted. However, due to the random nature of X-inactivation^48^, assessments of X-inactivation in blood samples may not accurately reflect this phenomenon in muscle tissue, which is pertinent to disease expression. With the expected increased use of and access to genetic testing, it will be important to develop a standardized strategy for reclassification of VUSs.

## Methods

### Clinically identified cohort

As part of an international collaboration, patients with a confirmed diagnosis of GSD IXα1 identified clinically were included in this study. All research procedures were conducted in accordance with all relevant ethical regulations including the principles of the Declaration of Helsinki. Participants able to provide written informed consent were enrolled in a longitudinal, retrospective natural history study approved by Duke University Health System Institutional Review Board Pro00104116 (ClinicalTrials.gov Identifier: NCT04454216, registered 2020-07-01) and all available paper and electronic medical records were reviewed (CONSORT checklist provided in the Supplementary File). Additionally, de-identified data was obtained using a targeted, study-specific spreadsheet from collaborating institutions data. For de-identified data from the United Kingdom and Germany, approval was received by the respective ethics boards to share deidentified data outside the organizations. For de-identified data from the Netherlands, the Medical Ethical Committee of the University Medical Center Groningen (UMCG) stated that the Medical Research Involving Human Subjects Act is not applicable and that further study approval by the Medical Ethical Committee is not required for retrospective, non-interventional studies (METc 2019/119). The patients were classified in two categories based on the variant detected in *PHKA1*: (1) pathogenic or likely pathogenic, or (2) variant of uncertain significance (VUS) using American College of Medical Genetics and Genomics (ACMG) criteria accessed on the ClinVar^49^ and gnomAD databases^50^. When available, muscle histology slides were reviewed by a neuropathologist specializing in muscle pathology.

### All of Us cohort

The All of Us Research Program is an NIH-led precision medicine resource which contains genome sequencing data and paired comprehensive clinical data on a large, diverse cohort (≥18 years of age) from the United States which permitted the exploration of phenotypic and genomic data^51^. The All of Us cohort builder tool was used to select individuals in the All of Us dataset containing annotated genomic data. The following loss-of-function variants in *PHKA1* are deletions or frameshift variants that were annotated in the All of Us dataset as “pathogenic” or “likely pathogenic”: X-72582562-C-A; X-72657614-G-A; X-72609623-C-T; X-72605280-G-A; X-72627005-G-A; X-72619228-G-A; X-72609625-CAG-C; or X-72656122-G-A. Due to the variability in clinical presentation and lack of functional testing, we opted for a selection of pathogenic/likely pathogenic variants only, not covering the VUSs in our study of this dataset.

Python scripts through the All of Us cloud computing environment were used to analyze clinical data, including diagnosis codes, laboratory measurements, and surveys. History of chronic pain was evaluated by searching for diagnoses related to pain (Athena concept ID 4329041) and hits from this concept code were reviewed to remove non-muscle related pain diagnoses, such as abdominal pain and migraines. We were granted an exception to the Data and Statistics Dissemination Policy from the *All of Us* Resource Access Board to display information from participant counts <20.

## Supplementary information

Supplementary Information

## Data Availability

All data generated or analyzed during this study are included in this published article and its supplementary files. Requests for additional data should be directed to the corresponding author.

## Code availability

The underlying code generated for the All of Us Research Program analysis is available to registered users of the All of Us Research Hub and accessible through the All of Us workbench at https://workbench.researchallofus.org/.

## References

[1] Candela, E. et al. Understanding glycogen storage disease type IX: a systematic review with clinical focus-why it is not benign and requires vigilance. *Genes* **16**, 10.3390/genes16050584 (2025). 10.3390/genes16050584 PMC12111550 40428406

[2] Kishnani, P. S. et al. Diagnosis and management of glycogen storage diseases type VI and IX: a clinical practice resource of the American College of Medical Genetics and Genomics (ACMG). *Genet. Med.* **21**, 772–789 (2019). 30659246 10.1038/s41436-018-0364-2

[3] Abarbanel, J. M. et al. Adult muscle phosphorylase “b” kinase deficiency. *Neurology* **36**, 560–562 (1986). 3083284 10.1212/wnl.36.4.560

[4] Carrier, H. et al. Myopathic evolution of an exertional muscle pain syndrome with phosphorylase b kinase deficiency. *Acta Neuropathol.* **81**, 84–88 (1990). 2128163 10.1007/BF00662642

[5] Ohtani, Y. et al. Infantile glycogen storage myopathy in a girl with phosphorylase kinase deficiency. *Neurology* **32**, 833–838 (1982). 6285226 10.1212/wnl.32.8.833

[6] Iwamasa, T. et al. Myopathy due to glycogen storage disease: pathological and biochemical studies in relation to glycogenosome formation. *Exp. Mol. Pathol.* **38**, 405–420 (1983). 6574020 10.1016/0014-4800(83)90080-1

[7] Bührer, C. et al. Fetal-onset severe skeletal muscle glycogenosis associated with phosphorylase-b kinase deficiency. *Neuropediatrics* **31**, 104–106 (2000). 10832587 10.1055/s-2000-7482

[8] Sahin, G. et al. Infantile muscle phosphorylase-b-kinase deficiency. A case report. *Neuropediatrics* **29**, 48–50 (1998). 9553951 10.1055/s-2007-973535

[9] Shin, Y. S. et al. Fatal arthrogryposis with respiratory insufficiency: a possible case of muscle phosphorylase b-kinase deficiency. *J. Inherit. Metab. Dis.* **17**, 153–155 (1994). 8051930 10.1007/BF00735424

[10] Danon, M. J., Carpenter, S., Manaligod, J. R. & Schliselfeld, L. H. Fatal infantile glycogen storage disease: deficiency of phosphofructokinase and phosphorylase b kinase. *Neurology* **31**, 1303–1307 (1981). 6213881 10.1212/wnl.31.10.1303

[11] Bak, H., Cordato, D., Carey, W. F. & Milder, D. Adult-onset exercise intolerance due to phosphorylase b kinase deficiency. *J. Clin. Neurosci.* **8**, 286–287 (2001). 11386811 10.1054/jocn.1999.0230

[12] Laforêt, P. et al. Exercise intolerance caused by muscular phosphorylase kinase deficiency. Contribution of in vivo metabolic studies. *Rev. Neurol.* **152**, 458–464 (1996). 8944243

[13] Wilkinson, D. A. et al. Clinical and biochemical features of 10 adult patients with muscle phosphorylase kinase deficiency. *Neurology* **44**, 461–466 (1994). 8145916 10.1212/wnl.44.3_part_1.461

[14] Andersen, A. G. et al. No effect of oral sucrose or IV glucose during exercise in phosphorylase b kinase deficiency. *Neuromuscul. Disord.* **30**, 340–345 (2020). 32303402 10.1016/j.nmd.2020.02.008

[15] Bisciglia, M. et al. A novel PHKA1 mutation associating myopathy and cognitive impairment: expanding the spectrum of phosphorylase kinase b (PhK) deficiency. *J. Neurol. Sci.* **424**, 117391 (2021). 33799212 10.1016/j.jns.2021.117391

[16] Bruno, C. et al. A splice junction mutation in the alpha(M) gene of phosphorylase kinase in a patient with myopathy. *Biochem. Biophys. Res. Commun.* **249**, 648–651 (1998). 9731190 10.1006/bbrc.1998.9211

[17] Burwinkel, B. et al. Muscle glycogenosis with low phosphorylase kinase activity: mutations in PHKA1, PHKG1 or six other candidate genes explain only a minority of cases. *Eur. J. Hum. Genet.* **11**, 516–526 (2003). 12825073 10.1038/sj.ejhg.5200996

[18] Echaniz-Laguna, A. et al. Muscle phosphorylase b kinase deficiency revisited. *Neuromuscul. Disord.* **20**, 125–127 (2010). 20080404 10.1016/j.nmd.2009.11.004

[19] Huang, K. et al. Expanding the clinicopathological-genetic spectrum of glycogen storage disease type IXd by a Chinese neuromuscular center. *Front. Neurol.* **13**, 945280 (2022). 36034300 10.3389/fneur.2022.945280 PMC9406516

[20] Li, H., Xue, Y., Yu, J., Guo, S. & Liu, C. An unusual case of recurrent episodes of muscle weakness: co-occurrence of Andersen-Tawil syndrome and glycogen storage disease type IXd. *Neuromuscul. Disord.* **30**, 562–565 (2020). 32660786 10.1016/j.nmd.2020.06.006

[21] Mori-Yoshimura, M. et al. A 78-year-old Japanese male with late-onset PHKA1-associated distal myopathy: Case report and literature review. *Neuromuscul. Disord.* **32**, 769–773 (2022). 35710611 10.1016/j.nmd.2022.05.010

[22] Munekane, A. et al. Maximal multistage shuttle run test-induced myalgia in a patient with muscle phosphorylase B kinase deficiency. *Intern. Med.* **61**, 1241–1245 (2022). 34615823 10.2169/internalmedicine.8137-21 PMC9107984

[23] Ørngreen, M. C. et al. Is muscle glycogenolysis impaired in X-linked phosphorylase b kinase deficiency?. *Neurology* **70**, 1876–1882 (2008). 18401027 10.1212/01.wnl.0000289190.66955.67

[24] Picillo, E., Onore, M. E., Passamano, L., Nigro, V. & Politano, L. A rare co-occurrence of phosphorylase kinase deficiency (GSD type IXd) and alpha-glycosidase deficiency (GSD Type II) in a 53-year-old man presenting with an atypical glycogen storage disease phenotype. *Acta Myol.* **43**, 21–26 (2024). 38586167 10.36185/2532-1900-411 PMC10997040

[25] Preisler, N. et al. Muscle phosphorylase kinase deficiency: a neutral metabolic variant or a disease?. *Neurology* **78**, 265–268 (2012). 22238410 10.1212/WNL.0b013e31824365f9

[26] Wang, C.-C. et al. Novel PHKA1 mutation in glycogen storage disease type IXD with typical myotonic discharges. *CNS Neurosci. Therapeutics* **28**, 1895–1897 (2022). 10.1111/cns.13939 PMC9532901 35957617

[27] Wehner, M., Clemens, P. R., Engel, A. G. & Kilimann, M. W. Human muscle glycogenosis due to phosphorylase kinase deficiency associated with a nonsense mutation in the muscle isoform of the alpha subunit. *Hum. Mol. Genet* **3**, 1983–1987 (1994). 7874115 10.1093/hmg/3.11.1983

[28] Wuyts, W. et al. Myopathy and phosphorylase kinase deficiency caused by a mutation in the PHKA1 gene. *Am. J. Med Genet. A***133a**, 82–84 (2005). 15637709 10.1002/ajmg.a.30517

[29] Clemens, P. R., Yamamoto, M. & Engel, A. G. Adult phosphorylase b kinase deficiency. *Ann. Neurol.* **28**, 529–538 (1990). 2252364 10.1002/ana.410280410

[30] Tarnopolsky, M. Exercise testing in metabolic myopathies. *Phys. Med. Rehabil. Clin. N. Am.* **23**, 173–186 (2012). xii. 22239882 10.1016/j.pmr.2011.11.011

[31] Elliot, D. L. et al. Metabolic myopathies: evaluation by graded exercise testing. *Medicine* **68**, 163–172 (1989). 2716515

[32] Kitaoka, Y. McArdle disease and exercise physiology. *Biology* **3**, 157–166 (2014). 24833339 10.3390/biology3010157 PMC4009758

[33] Vissing, J. & Haller, R. G. The effect of oral sucrose on exercise tolerance in patients with McArdle’s disease. *N. Engl. J. Med.* **349**, 2503–2509 (2003). 14695410 10.1056/NEJMoa031836

[34] Lucia, A. et al. Clinical practice guidelines for glycogen storage disease V & VII (McArdle disease and Tarui disease) from an international study group. *Neuromuscul. Disord.* **31**, 1296–1310 (2021). 34848128 10.1016/j.nmd.2021.10.006

[35] Koch, R. L. et al. Diagnosis and management of glycogen storage disease type IV, including adult polyglucosan body disease: A clinical practice resource. *Mol. Genet. Metab.* **138**, 107525 (2023). 36796138 10.1016/j.ymgme.2023.107525

[36] Hagemans, M. L. et al. Fatigue: an important feature of late-onset Pompe disease. *J. Neurol.* **254**, 941–945 (2007). 17351726 10.1007/s00415-006-0434-2 PMC2779379

[37] Yang, X., Zhu, M., Lu, X., Wang, Y. & Xiao, J. Architecture and activation of human muscle phosphorylase kinase. *Nat. Commun.* **15**, 2719 (2024). 38548794 10.1038/s41467-024-47049-2 PMC10978961

[38] Ma, R. et al. Molecular basis for the regulation of human phosphorylase kinase by phosphorylation and Ca2+. *Nat. Commun.* **16**, 3020 (2025). 40148320 10.1038/s41467-025-58363-8 PMC11950179

[39] Hargreaves, M. & Spriet, L. L. Skeletal muscle energy metabolism during exercise. *Nat. Metab.* **2**, 817–828 (2020). 32747792 10.1038/s42255-020-0251-4

[40] Hannah, W. B. et al. Glycogen storage diseases. *Nat. Rev. Dis. Prim.* **9**, 46 (2023). 37679331 10.1038/s41572-023-00456-z

[41] Raben, N., Wong, A., Ralston, E. & Myerowitz, R. Autophagy and mitochondria in Pompe disease: Nothing is so new as what has long been forgotten. *Am. J. Med. Genet. Part C: Semin. Med. Genet.* **160C**, 13–21 (2012). 22253254 10.1002/ajmg.c.31317 PMC3265635

[42] Schoser, B. Pompe disease: what are we missing? *Ann. Transl. Med.* **7**, 292 (2019). 31392204 10.21037/atm.2019.05.29 PMC6642932

[43] Schneider, A., Davidson, J. J., Wüllrich, A. & Kilimann, M. W. Phosphorylase kinase deficiency in I-strain mice is associated with a frameshift mutation in the alpha subunit muscle isoform. *Nat. Genet.* **5**, 381–385 (1993). 8298647 10.1038/ng1293-381

[44] Mefford, A. M., Ayers, C. C., Rowland, N. S. & Rice, N. A. The phka1 deficient I/LnJ mouse exhibits endurance exercise deficiency with no compensatory changes in glycolytic gene expression. *Open J. Mol. Integr. Physiol.* **03**, 87–94 (2013).

[45] Haller, R. G. & Vissing, J. Spontaneous “second wind” and glucose-induced second “second wind” in McArdle disease: oxidative mechanisms. *Arch. Neurol.* **59**, 1395–1402 (2002). 12223025 10.1001/archneur.59.9.1395

[46] Løkken, N. et al. Can a modified ketogenic diet be a nutritional strategy for patients with McArdle disease? Results from a randomized, single-blind, placebo-controlled, cross-over study. *Clin. Nutr.* **42**, 2124–2137 (2023). 37769369 10.1016/j.clnu.2023.09.006

[47] Andersen, S. T. & Vissing, J. Carbohydrate- and protein-rich diets in McArdle disease: effects on exercise capacity. *J. Neurol. Neurosurg. Psychiatry* **79**, 1359–1363 (2008). 19010947 10.1136/adc.2008.146548

[48] Juchniewicz, P. et al. Dosage compensation in females with X-linked metabolic disorders. *Int. J. Mol. Sci.* **22**, 10.3390/ijms22094514 (2021). 10.3390/ijms22094514 PMC8123450 33925963

[49] Landrum, M. J. et al. ClinVar: improving access to variant interpretations and supporting evidence. *Nucleic Acids Res.* **46**, D1062–D1067 (2017). 10.1093/nar/gkx1153 PMC5753237 29165669

[50] Chen, S. et al. A genomic mutational constraint map using variation in 76,156 human genomes. *Nature* **625**, 92–100 (2024). 38057664 10.1038/s41586-023-06045-0 PMC11629659

[51] Bick, A. G. et al. Genomic data in the All of Us Research Program. *Nature* **627**, 340–346 (2024). 38374255 10.1038/s41586-023-06957-x PMC10937371

[52] Savostyanov, K. V. et al. New method for molecular genetic diagnosis of glycogen storage disease in Russian patients using next-generation sequencing (NGS). *J. Inher Metab. Dis.* **39**, S203–S204 (2016).

