# Author Correction: PHKA1-associated phosphorylase kinase deficiency: a monogenic disorder of exercise intolerance and myalgia

**DOI:** 10.1038/s41525-025-00544-x

**Published:** 2025-12-30

**Authors:** Rebecca L. Koch, Angie H. Fares, Benjamin T. Cocanougher, Jamie Lim, Andrea B. Haijer-Schreuder, Terry G. J. Derks, Sarah C. Grünert, Reena Sharma, Karra A. Jones, Priya S. Kishnani

**Affiliations:** 1 Division of Medical Genetics, Department of Pediatrics, Duke University Medical Center, Durham, NC USA; 2 Division of Metabolic Diseases, Beatrix Children’s Hospital, University Medical Center Groningen, Groningen, the Netherlands; 3 Department of General Pediatrics, Adolescent Medicine and Neonatology, Medical Center - University of Freiburg, Faculty of Medicine, Freiburg, Germany; 4 The Mark Holland Metabolic Unit, Adult Inherited Metabolic Diseases Department, Salford Royal NHS Foundation Trust, Salford, UK; 5 Department of Pathology, Division of Neuropathology, Duke University, Durham, NC USA

**Keywords:** Metabolic disorders, Medical genetics, Genetics research

Correction to: *npj Genomic Medicine* 10.1038/s41525-025-00527-y, published online 10 November 2025

In this article the citation information for Figure 1 was incorrectly given as #24 but should have been #37. The original article has been corrected.

