# Supplementary material for: PHKA1-associated phosphorylase kinase deficiency: a monogenic disorder of exercise intolerance and myalgia
Source: NPJ Genom Med. 2025 Nov 10;10:71. doi: 10.1038/s41525-025-00527-y (PMC12603314; doi:10.1038/s41525-025-00527-y)
Supplement: Supplementary file 1 — Supplementary Information [file 41525_2025_527_MOESM1_ESM.pdf]

Supplementary Table S1. Muscle histology findings from individuals with GSD IXα1.

Results from pathology review of muscle biopsies from patients with a pathogenic/likely pathogenic variant that underwent a muscle biopsy as part of their routine clinical care compared to that of patients previously reported in the literature. Histology slides from patients 2 and 8 were available for review by a neuropathologist specializing in muscle pathology, all other findings come from the pathology report. Abbreviations: +, present; -, not present; IVFS, increased variation in fiber size; ND, no data/not reported.

ID	Biopsy site	Glycogen deposition	IVFS	Additional Findings
Patient 2	Left vastus lateralis	+	+	Vacuoles on acid phosphatase and by electron microscopy
Patient 7	Right tibialis anterior	+	+	Type I fiber predominance, vacuoles on acid phosphatase
Patient 8	Right vastus lateralis	+	+	Vacuoles on acid phosphatase and by electron microscopy
Patient 9	Right vastus lateralis	-	+	Type I fiber predominance
L1	Unspecified	+	ND	
L2	Unspecified	+	ND	
L4	Unspecified	+	ND	
L5	Unspecified	+	ND	
L7	Unspecified	+	ND	
L8	Unspecified	+	+	Type I fiber predominance, scattered angular fibers, regenerative fibers
L9	Unspecified	+	ND	
L10	Unspecified	+	+	Scattered necrotic fibers, regeneration, slight hyperplasia of connective tissue, vacuoles
L11	Unspecified	+	ND	
L12	Unspecified	+	ND	
L13	Unspecified	+	ND	
L14	Left anterior tibialis	+	+	Vacuoles without rims and ring fibers were scattered, destruction of sarcomeres
L15	Unspecified	+	ND	
L16	Unspecified	+	+	Scattered atrophic fibers, slight hyperplasia of connective tissue, vacuoles (HE staining), myofibrillar network disarray, RRF (modified Gömöri staining), RBF (SDH staining)
L17	Unspecified	+	ND	
L18	Unspecified	-	+	Type I fiber predominance, increased number of nuclei

Supplementary Table S2. Summary of findings in patients with a VUS.

All reported variants are missense VUS (PM2, PP3) according to ACMG classification. Patients were ordered according to the age at which symptoms first presented. Ages are provided in years. The highest creatine kinase (CK) level detected since presentation is reported and the upper limit of normal (N) was considered 310 U/L. Allele count and frequency were collected from the gnomAD database.[1] Abbreviations: WES, whole exome sequencing; WGS, whole genome sequencing.

ID	Sex	Location of care	Age at onset	Age at last follow up	GSD IXα1-related chief complaints	*PHKA1* allele			Allele count	Allele frequency	Genetic testing	CK
						Variant	Location	Type				
Patient 11	M	Canada	2.4	3.9	Exercise intolerance, gross motor delays	c.1745T>C (p.Ile582Thr)	Exon 17	Missense	6	5E-06	WES[a]	2.4N
Patient 12	M	USA	4.2	13.4	Lower extremity weakness, fatigue	c.849C>G (p.Ile283Met)	Exon 8	Missense	Not found on gnomAD	Not found on gnomAD	WES[b]	N
Patient 13	F	USA	14	21	Exercise intolerance, myalgia, lower extremity weakness	c.1079T>C (p.Ile360Thr)	Exon 11	Missense	11	1E-05	Targeted gene testing	N
Patient 14	M	USA	38	46	Myalgia, muscle weakness, dysphagia	c.1079T>C (p.Ile360Thr)	Exon 11	Missense	11	1E-05	WES	14.6N

[a]A variant of uncertain significance in *RYR1* at c.3253G>A (p.Gly1085Ser) was also identified and has been associated with autosomal dominant congenital myopathies, often with type I fiber predominance.
[b]A likely pathogenic variant in *EXT2* at c.1719G>A (p.Trp573Ter) and a variant of uncertain significance in *COL11A2* at c.3311A>C (p.His1104Pro) were also identified and have been associated with hereditary multiple exostoses type II and disorders related to collagen type XI, respectively.

References:

1. Chen S, Francioli LC, Goodrich JK, et al. A genomic mutational constraint map using variation in 76,156 human genomes. Nature 2024;625:92-100.

Patient 11

Patient 11 presented at age 2 years with an inability to run or jump. Physical examination done at the time showed bilateral calf hypertrophy in the setting of an elevated CK level (754 U/L) and a normal EMG of the right gastrocnemius. A biopsy of the right vastus lateralis detected no significant variation in fiber size with fairly normal findings on H&E stain. COX and SDH stains highlight course mitochondrial staining within myofibers, aggregating around linear glycogen deposition visible in myofibers on PAS stain. Gomori trichrome stain showed a similar linear pattern of abnormal red granularity to what was seen on COX, SDH, and PAS stains. Toluidine blue staining of resin sections showed large aggregates of glycogen in the subsarcolemmal space, with some in the center of the sarcoplasm in fibers oriented longitudinally. Some of the excess glycogen was associated with autophagic vacuolar pathology. Electron microscopic images confirmed the presence of increased predominately free glycogen in between sarcomeres and in the subsarcolemmal space. Rare foci showed membrane-bound glycogen. Autophagic vacuolar pathology was multifocal. Significant aggregates of abnormal mitochondria were seen directly associated with excess glycogen. Mitochondria showed enlargement, rounded electron dense inclusions, course cristae, and rare paracrystalline inclusions. At age 3 years, symptoms progressed to include exercise intolerance. WES revealed a missense VUS in *PHKA1* (c.1745T>C, p.Ile582Thr; confirmed to be maternally inherited) and another missense VUS in *RYR1* (c.3253G>A p.Gly1085Ser). Physical exam done at the same age revealed an exacerbation in calves and quadriceps hypertrophy with lower extremity muscle wasting. He was following a high-protein diet with pre-exercise carbohydrates with no symptomatic benefits. Patient 11 is currently 5 years old with persistent exercise intolerance, more pronounced on moderate to intense physical activity.

Patient 12

Patient 12 is a 15-year-old Caucasian male with a past medical history significant for Tourette and autism spectrum disorder (ASD). At the age of 4 years, he developed lethargy peaking after half a day at school or 30 minutes of activity. Symptoms progressed at the age of 6 years to include exercise intolerance with

episodes of falling on exertion, in the setting of an elevated CK levels (624 U/L). By the age of 10 years, his CK levels normalized to 151 U/L but his myopathic presentation persisted. WES revealed a VUS in *PHKA1* (c.849C>G, p.Ile283Met), confirmed to be maternally inherited. At age 12 years, patient 12's exercise intolerance significantly progressed to muscle weakness and cramping, particularly in the cervical, shoulder, and hip regions, precipitated by movements like reaching or door opening. He increasingly relied on a wheelchair for mobility. Gowers' sign was negative and EMG studies yielded unremarkable results. He was following a regular diet supplemented with carnitine with no notable improvement. He is currently 15 years old, receiving multidisciplinary physical and occupational therapy to address the patient's clinical presentation.

Patient 13

Patient 13 is the daughter of patient 14 and a Caucasian female whose medical history includes a diagnosis of pervasive developmental disorder, attention deficit disorder (ADD/ADHD) and delays in language milestones. At age 14 years, she started experiencing leg weakness after strenuous physical activities, often requiring assistance to ambulate. An EMG and nerve conduction study (NCS) did not reveal any abnormalities. At age 16 years, her symptoms progressed to include exercise intolerance and lethargy on moderate activities like running or jumping. Due to the resemblance of her symptoms to her father (patient 14), targeted genetic testing was performed and revealed a heterozygous VUS in *PHKA1 (*c.1079T>C, p.Ile360Thr), confirmed to be paternally inherited. Distal lower extremity muscle fasciculations were reported at the same time, which are not known to be related to the variant in *PHKA1*. Upon identification of the *PHKA1* variant, patient 13 adopted a high-protein, low-carbohydrate diet 13 which reportedly helped decrease myalgia. CK levels were within normal limits (57 U/L) at age 16 years. She has found relief through physical therapy sessions conducted twice a week. She was 21 years old at her last follow-up, still reporting myalgia along with hip and knee pain and a tendency to bruise easily. Bony pains and bruising tendency are not known symptoms of GSD IXα1. Given a positive anti-nuclear antibody (ANA) screen

detected at last follow-up, additional investigations are currently underway to assess the possibility of an autoimmune condition.

Patient 14

Patient 14 is a Caucasian male who started experiencing lower extremity cramps, and fatigue on exertion at the age of 38 years. Dysphagia was also reported with no episodes of aspiration. CK levels were found to be elevated (4517 U/L) at the time in the setting of normal EMG results. At the same age, a muscle biopsy of the left quadriceps muscle was performed with no specific histopathologic changes except for mild fiber atrophy, mild mitochondrial, glycogen, and lipid changes, and evidence of minimal active denervation, which prompted genetic investigation. WES revealed a missense VUS in *PHKA1* (c.1079T>C, p.Ile360Thr) and a microarray identified a copy number variation of unknown clinical significance on chromosome 2p12. Presumably unexplained by the variant in *PHKA1,* he reported experiencing paresthesia, pain, and weakness in the left side of the tongue, leading to slurred speech in the evenings. At age 40 years, symptoms progressed to persistent myalgia, stiffness, and lethargy. Gowers' sign was positive, and CK levels were normal at 103 U/L. He reported symptomatic exacerbation with carbohydrate consumption and improvement on a high-protein diet. Physical examination at that time was significant for the presence of angiokeratomas and testing revealed a fatty spleen, mild left ventricular hypertrophy, and hypertriglyceridemia in the setting of a BMI of 33 kg/m2. α-galactosidase levels were normal – ruling out Fabry disease. During his last follow-up at the age of 46 years, he reported persistent myopathic symptoms.

Supplementary Figure S1. Identification of individuals with a pathogenic (PATH) or likely pathogenic (LP) variant in *PHKA1* in the All of Us dataset.

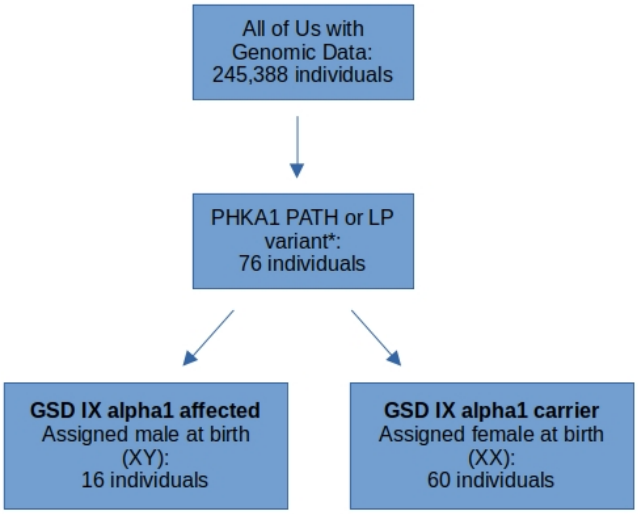

Supplementary Figure S2. Number of individuals assigned male at birth with available whole genome sequencing short read data (total count: 94,756).

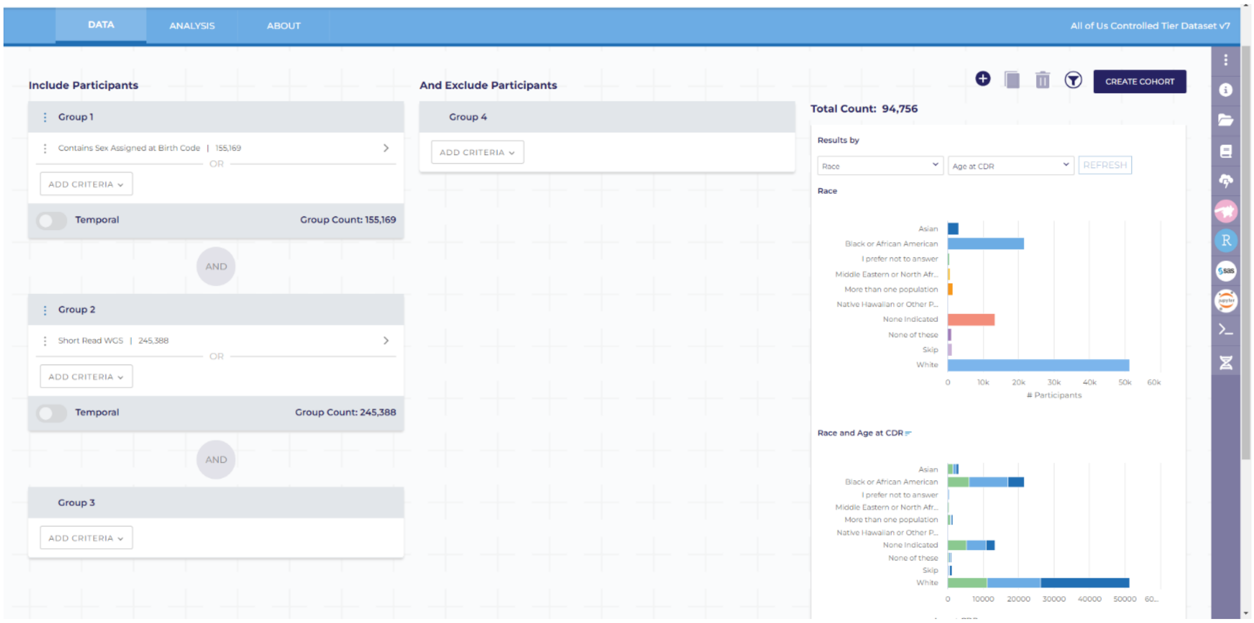

Supplementary Figure S3. Number of individuals assigned female at birth with available whole genome sequencing short read data (total count: 145,563).

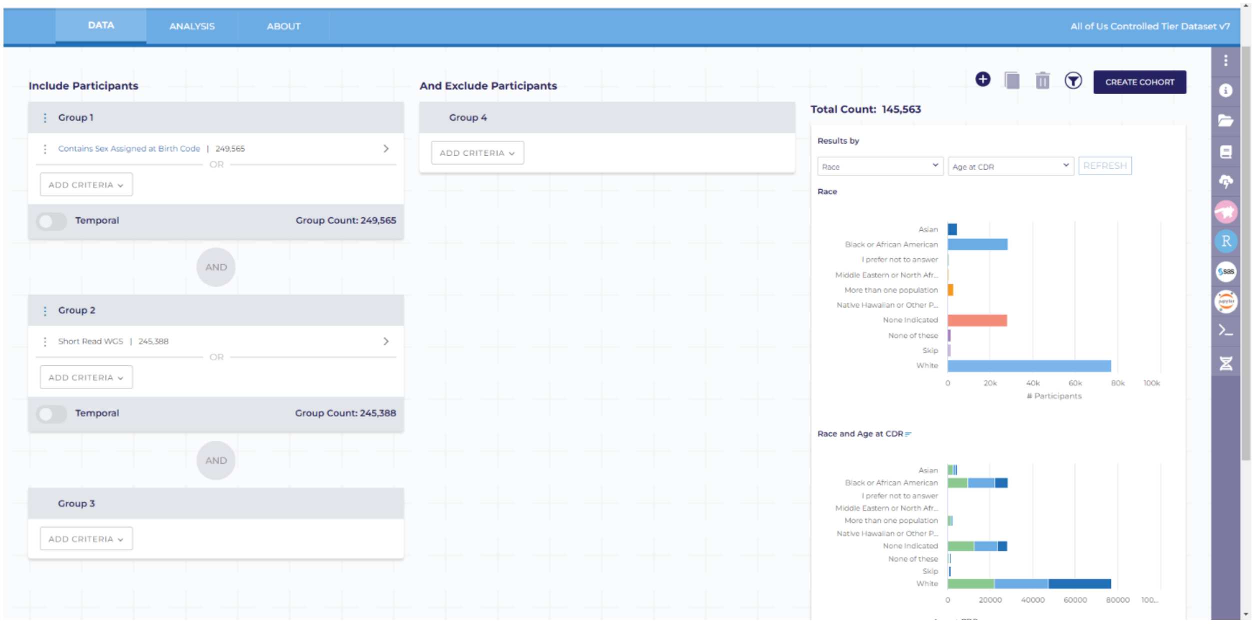

Supplementary Figure S4. Selected concepts from "condition" consistent with a GSD IXα1.

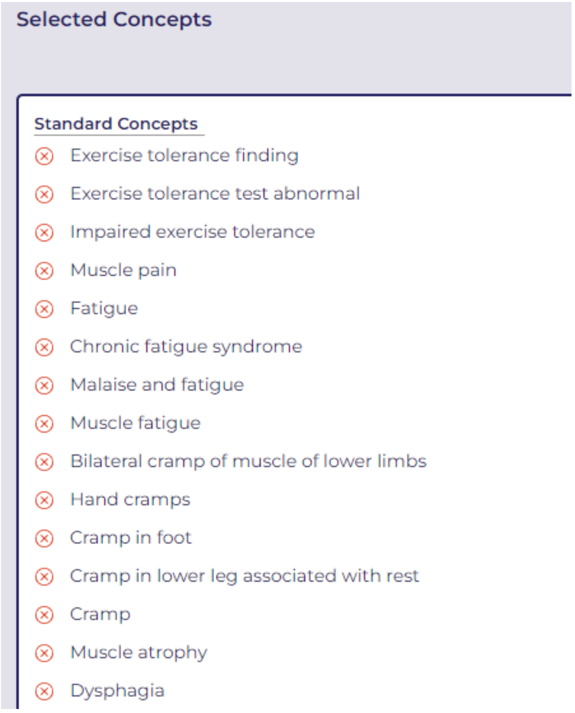

Section/topic	No	CONSORT 2025 checklist item description	Reported on page no.
**Title and abstract**			
Title and structured abstract	1a	Identification as a randomised trial	Not applicable
	1b	Structured summary of the trial design, methods, results, and conclusions	Abstract (2)
**Open science**			
Trial registration	2	Name of trial registry, identifying number (with URL) and date of registration	Methods (15)
Protocol and statistical analysis plan	3	Where the trial protocol and statistical analysis plan can be accessed	Methods (15)
Data sharing	4	Where and how the individual de-identified participant data (including data dictionary), statistical code and any other materials can be accessed	Data availability (16)
Funding and conflicts of interest	5a	Sources of funding and other support (eg, supply of drugs), and role of funders in the design, conduct, analysis and reporting of the trial	Acknowledgme nts (17)
	5b	Financial and other conflicts of interest of the manuscript authors	Competing Interests (17)
**Introduction**			
Background and rationale	6	Scientific background and rationale	Introduction (3)
Objectives	7	Specific objectives related to benefits and harms	Introduction (3)
**Methods**			
Patient and public involvement	8	Details of patient or public involvement in the design, conduct and reporting of the trial	Methods (15)
Trial design	9	Description of trial design including type of trial (eg, parallel group, crossover), allocation ratio, and framework (eg, superiority, equivalence, non-inferiority, exploratory)	Methods (15)
Changes to trial protocol	10	Important changes to the trial after it commenced including any outcomes or analyses that were not prespecified, with reason	Not applicable
Trial setting	11	Settings (eg, community, hospital) and locations (eg, countries, sites) where the trial was conducted	Methods (15)
Eligibility criteria	12a	Eligibility criteria for participants	Methods (15)
	12b	If applicable, eligibility criteria for sites and for individuals delivering the interventions (eg, surgeons, physiotherapists)	Not applicable
Intervention and comparator	13	Intervention and comparator with sufficient details to allow replication. If relevant, where additional materials describing the intervention and comparator (eg, intervention manual) can be accessed	Not applicable
Outcomes	14	Prespecified primary and secondary outcomes, including the specific measurement variable (eg, systolic blood pressure), analysis metric (eg, change from baseline, final value, time to event), method of aggregation (eg, median, proportion), and time point for each outcome	Not applicable
Harms	15	How harms were defined and assessed (eg, systematically, non-systematically)	Not applicable
Sample size	16a	How sample size was determined, including all assumptions supporting the sample size calculation	Not applicable
	16b	Explanation of any interim analyses and stopping guidelines	Not applicable
Randomisation:			Not applicable
Sequence generation	17a	Who generated the random allocation sequence and the method used	
	17b	Type of randomisation and details of any restriction (eg, stratification, blocking and block size)	Not applicable

			Reported on page no.
Allocation concealment mechanism	18	Mechanism used to implement the random allocation sequence (eg, central computer/telephone; sequentially numbered, opaque, sealed containers), describing any steps to conceal the sequence until interventions were assigned	Not applicable
Implementation	19	Whether the personnel who enrolled and those who assigned participants to the interventions had access to the random allocation sequence	Not applicable
Blinding	20a	Who was blinded after assignment to interventions (eg, participants, care providers, outcome assessors, data analysts)	Not applicable
	20b	If blinded, how blinding was achieved and description of the similarity of interventions	Not applicable
Statistical methods	21a	Statistical methods used to compare groups for primary and secondary outcomes, including harms	Not applicable
	21b	Definition of who is included in each analysis (eg, all randomised participants), and in which group	Not applicable
	21c	How missing data were handled in the analysis	Not applicable
	21d	Methods for any additional analyses (eg, subgroup and sensitivity analyses), distinguishing prespecified from post hoc	Not applicable
**Results**			
Participant flow, including flow diagram	22a	For each group, the numbers of participants who were randomly assigned, received intended intervention, and were analysed for the primary outcome	Not applicable
	22b	For each group, losses and exclusions after randomisation, together with reasons	Not applicable
Recruitment	23a	Dates defining the periods of recruitment and follow-up for outcomes of benefits and harms	Not applicable
	23b	If relevant, why the trial ended or was stopped	Not applicable
Intervention and comparator delivery	24a	Intervention and comparator as they were actually administered (eg, where appropriate, who delivered the intervention/comparator, how participants adhered, whether they were delivered as intended (fidelity))	Not applicable
	24b	Concomitant care received during the trial for each group	Not applicable
Baseline data	25	A table showing baseline demographic and clinical characteristics for each group	Table 1 (25)
Numbers analysed, outcomes and estimation	26	For each primary and secondary outcome, by group:<br>● the number of participants included in the analysis<br>● the number of participants with available data at the outcome time point<br>● result for each group, and the estimated effect size and its precision (such as 95% confidence interval)<br>● for binary outcomes, presentation of both absolute and relative effect size	Not applicable
Harms	27	All harms or unintended events in each group	Not applicable
Ancillary analyses	28	Any other analyses performed, including subgroup and sensitivity analyses, distinguishing pre-specified from post hoc	Not applicable
**Discussion**			
Interpretation	29	Interpretation consistent with results, balancing benefits and harms, and considering other relevant evidence	Discussion (9-14)
Limitations	30	Trial limitations, addressing sources of potential bias, imprecision, generalisability, and, if relevant, multiplicity of analyses	Discussion (15)

Citation: Hopewell S, Chan AW, Collins GS, Hróbjartsson A, Moher D, Schulz KF, et al. CONSORT 2025 Statement: updated guideline for reporting randomised trials. BMJ. 2025; 388:e081123. https://dx.doi.org/10.1136/bmj-2024-081123
© 2025 Hopewell et al. This is an Open Access article distributed under the terms of the Creative Commons Attribution License (https://creativecommons.org/licenses/by/4.0/), which permits unrestricted use, distribution, and reproduction in any medium, provided the original work is properly cited.

*We strongly recommend reading this statement in conjunction with the CONSORT 2025 Explanation and Elaboration and/or the CONSORT 2025 Expanded Checklist for important clarifications on all the items. We also recommend reading relevant CONSORT extensions. See www.consort-spirit.org.
